# Impact of Military Service in Vietnam on Coping and Health Behaviors of Aging Veterans During the COVID-19 Pandemic

**DOI:** 10.3389/fpubh.2021.809357

**Published:** 2022-01-17

**Authors:** Jeanne M. Stellman, Steven D. Stellman, Avron Spiro, Anica Pless Kaiser, Brian N. Smith

**Affiliations:** ^1^Department of Health Policy and Management, Mailman School of Public Health, Columbia University, New York, NY, United States; ^2^Department of Epidemiology, Mailman School of Public Health, Columbia University, New York, NY, United States; ^3^Massachusetts Veterans Epidemiology Research and Information Center (MAVERIC), VA Boston Healthcare System and Departments of Epidemiology and Psychiatry, Boston University Schools of Public Health and Medicine, Boston, MA, United States; ^4^National Center for PTSD Behavioral Science Division at VA Boston Healthcare System and Department of Psychiatry, Boston University School of Medicine, Boston, MA, United States; ^5^National Center for PTSD Women's Health Sciences Division, VA Boston Healthcare System and Department of Psychiatry, Boston University School of Medicine, Boston, MA, United States

**Keywords:** Vietnam veterans, COVID-19, PTSD, health behavior, combat, coping, late onset stress symptomatology, aging

## Abstract

Many Vietnam War veterans who experienced military trauma still exhibit PTSD symptomatology. Little is known about how new stressful situations, like the COVID-19 pandemic, affect previously traumatized people or whether they will react differently to them. We explore whether military combat experiences in Vietnam affect veterans' perceived abilities to cope with COVID-19 and whether current PTSD symptoms and later-adulthood reengagement with trauma memories are related to coping. We examine the extent that current PTSD symptoms and trauma reengagement relate to preventive practices. Participants were part of a randomly sampled cohort of American Legionnaires who responded to two previous surveys (1984, 1998), were born 1945-1953 and deployed to Vietnam 1963-1973, thus representing an aging veteran population. A survey supplement assessed coping with the pandemic and adherence to public health guidelines. The response rate was 74% (*N* = 507); 422 (61.6%) completed the COVID-19 supplement. Military experiences were found to affect coping with 41.4% reporting they affected ability to cope with COVID-19. Medium- and high-combat veterans were more likely to report that military experience affected coping than low-combat (OR 2.4, 95% CI 1.51–3.96; 2.6, 95% CI 1.41–4.61, respectively). Those with high PTSD scores had 7.7-fold (95% CI 4.3–13.17) increased likelihood of reporting that their coping was affected, compared to low-PTSD scorers. Few adopted social distancing (4%), staying at home (17%), or ceasing usual activities (32%); high-combat veterans were least likely to stay home. Veterans who practiced handwashing, sanitizer use, mask-wearing, and surface disinfection had significantly higher PTSD scores than those who did not. Veterans with higher scores on the LOSS-SF scale associated more reengagement with trauma memories and were more likely to engage in personal preventive strategies. Analysis of open-ended responses supported these findings. We conclude that fifty years after returning from Vietnam, PTSD scores were high for high-combat veterans, suggestive of PTSD diagnosis. Military experiences affected coping with COVID both positively and negatively, and may have helped instill useful personal health behaviors. Veterans, especially those with PTSD symptomatology, may have special needs during stressful times, like the COVID-19 pandemic, affecting compliance with recommended practices, as well as their overall health and well-being.

## Introduction

Early in the COVID-19 pandemic, Gerber (1), an internist and expert in veterans' health and trauma-informed care, warned of the special burden many Vietnam veterans who had experienced military trauma could face as members of an aging population. (In 2020, the median age of Vietnam veterans was 71) (2). She posited that a number of older veterans would view the COVID-19 pandemic through the lens of their prior wartime experiences and could be retraumatized. Indeed, Gerber described how one of her colleagues, a nurse who had served in Vietnam, “broke when she shared that stories of COVID-19 patients dying in isolation without family at the bedside brought back her time in a Vietnam field hospital caring for mortally wounded soldiers.” Retraumatization could be more likely if veterans were forced to become isolated from their usual social support networks by COVID restrictions and preventive practices.

A sizable fraction of the 6.3 million surviving American Vietnam veterans faced heavy combat during their deployment. The cohort from which the current study population was drawn, a random sample of 12,400 male American Legionnaires first assembled in 1983, includes many men who were exposed to combat: 38.1% and 19.4% of these men deployed to Vietnam experienced medium and heavy combat, respectively (3).

Posttraumatic stress disorder (PTSD) symptomatology can vary significantly among veterans with comparable combat experiences, and symptoms within an individual veteran often exhibit a varying course over their lifetime (4–7). PTSD symptoms can also be persistent. In 1998 we observed that 10.5% of our deployed Legionnaires continued to report severe symptoms of PTSD some 25 years after their return from Vietnam (8). Similar findings have been reported in other studies of aging veterans (9, 10). PTSD is also associated with use of maladaptive coping strategies (e.g., substance use, over-working) and poor health behaviors, such as smoking, physical inactivity, and medication nonadherence (11).

Combat veterans who exhibit more PTSD symptoms may have a reduced ability to cope with stressors associated with the COVID-19 pandemic. Sachs-Ericsson and co-workers showed that recent life events associated with PTSD were significantly affected by previous exposure to high levels of combat but not to low levels (12). Similar effects were seen in previously traumatized refugees who were experiencing new critical life events (13).

There also appears to be a relationship between PTSD symptoms and risk perception, with traumatized individuals with clinical or subthreshold levels of PTSD symptoms perceiving new stressors, unrelated to their original trauma, as more threatening (14). Perceptions of risk, in turn, have also been found to be related to health behaviors (15). With respect to COVID-19, a national survey in the United States found that those who perceived more risk were significantly more likely to engage in frequent handwashing and social distancing (16). It is thus reasonable to hypothesize that veterans with ongoing PTSD symptomatology could have enhanced perception of threats associated with the COVID-19 pandemic and that these perceptions might affect their adherence to recommended public health practices, as well as increase their current levels of distress. Further, those who had experienced military trauma could behave differently from those who did not. Indeed, Haderlein et al. reported that veterans with a clinical diagnosis of PTSD were more likely to receive a COVID-19 test, but were less likely to test positive, which raises the possibility that veterans with PTSD may be perceiving more risk and availing themselves more of testing opportunities (17).

Altered perceptions of risk can also have physiological effects. Experimental studies of undergraduates showed that PTSD symptoms were not only associated with current physical health (e.g., resting blood pressure and heart rate), but also with more negative appraisals of stressors that were presented to them. The more negative appraisals were also associated with increases in cardiovascular response, and threat appraisal was found to mediate the relationship between PTSD symptoms and blood pressure responses (18).

### Military Experiences and Subsequent Civilian Life

It is well established that exposure during young adulthood to trauma, such as military combat, increases the risk for development of PTSD symptoms. As veterans age, many also experience loss-related challenges of aging (e.g., bereavement, retirement, role transition and loss, physical and cognitive decline) which can invoke reminders of earlier traumatic events and “lead to increased reminiscence, and possibly distress, among Veterans who had previously dealt successfully with traumatic events” (19). Recent work on a sample of United States (US) veterans from multiple wartime eras found that these effects can be seen across the age spectrum and that pre-pandemic loneliness, depression/PTSD, mindfulness, and purpose in life were most strongly associated with resilience, suggesting that preexisting vulnerability factors, as well as resilience-promoting factors, may also help shape psychological adaptation to the pandemic (7). A study of veterans in the United Kingdom (UK) similarly observed exacerbation of previous mental health difficulties during the COVID-19 pandemic (20). Another study of UK veterans who had served in the recent Iraq/Afghanistan era found that those who entered the pandemic with existing mental health concerns may be particularly impacted by COVID-19 stressors, further underscoring that the impact of military experiences on coping with the COVID-19 pandemic are not limited to aging Vietnam veterans (21).

Research generally has emphasized negative outcomes associated with war, and especially for older veterans such as those in our study, overlooked processes of reengagement, and in many cases reconciliation with earlier military trauma (22). It is important to note that military service can also provide positive professional experiences, as well as lead to lifelong comradeship and social support networks. Sixsmith and co-workers' observations of British Second World War veterans' wartime and subsequent life experiences showed that for many, their military service provided an opportunity to learn to be more self-sufficient and disciplined and to inculcate habits of personal hygiene (23). Our work with American women who served in military and civilian capacities in Vietnam during the war found that they described many positive aspects of their experiences, such as their sense of camaraderie with their peers and the troops, their ability to meaningfully help others, and the personal and professional growth they experienced by being called upon to carry out work that often expanded their skill set. Had they not been deployed, they would not have experienced many of these opportunities. Many also established enduring friendships and joined organizations, such as The American Legion, related to their service (24).

In the present study we investigated the extent to which early-life military experiences in male US veterans who served in Southeast Asia during the Vietnam War affected their ability to cope with the current COVID-19 pandemic. Specifically, we studied the extent to which prior military experiences and their long-term sequelae, including PTSD symptomatology and later life reengagement with trauma memories, affected their capacity to cope with the pandemic as reflected, for example, in adoption of recommended social and personal protective health behaviors. We sought to answer three questions:

(1) Did military combat experiences in Vietnam affect veterans' perceived abilities to cope with the COVID-19 pandemic?

(2) Were current PTSD symptoms and later-adulthood reengagement with trauma memories related to coping?

(3) To what extent do current PTSD symptoms and trauma reengagement relate to adoption of preventive practices?

## Materials and Methods

The data were gathered in a long-term study of American Legionnaires of the Vietnam Era begun in 1983 (3, 8). The American Legion is the largest veterans' service organization in the US. From July through October 2020, we surveyed a subgroup of men who had responded to two earlier survey waves in 1984 and 1998 and who had been deployed to Vietnam (as opposed to having been stationed in the United States or elsewhere in the world). Fielding the present survey during the first summer of the COVID-19 pandemic provided a unique opportunity to investigate whether prior military experiences and combat exposure, as well as their current PTSD symptoms and later life reengagement with trauma memories, affected how these veterans perceived themselves to be coping with the COVID-19 pandemic.

The original cohort of male American Legionnaire veterans was randomly sampled in October 1983 from American Legion Post membership rosters in six States (Colorado, Indiana, Maryland, Minnesota, Ohio, Pennsylvania). The cohort comprised men who had served in the US armed services during the Vietnam era. Efforts were made to include sufficient numbers of men who had been deployed to Vietnam because many more US veterans had served elsewhere in the world during the era. Given the small numbers of women who served in Vietnam (believed to be fewer than 12,000), and their small numbers in Legion rosters, the survey was limited to men. We have reported elsewhere on experiences of female Vietnam veterans (25).

The baseline survey (Wave 1) was fielded in 1984 (3), and a second survey (Wave 2) in 1998 (26). The aims of the study were to obtain and analyze information on the personal, reproductive, family, physical and mental health of the veterans, and on health behaviors, such as smoking, drinking, and substance use, and to determine the extent to which these behaviors and outcomes varied with respect to combat exposure. An additional aim was to characterize the respondents' exposure to herbicides like Agent Orange—using previously developed techniques that have been extensively validated—and its relationship to health outcomes (27, 28). We also considered veterans' attitudes toward and perceptions of the Veterans Administration (VA) and their experiences with its facilities and programs, since the VA is charged with providing for the health and well-being of American veterans (29).

In 2020, we resurveyed a subgroup comprising 729 men who had responded to both previous waves of the survey and, following extensive searches of public records to determine vital status of the original cohort, were believed to be still living. For this pilot survey (Wave 3), we limited the sample to men born 1945-1953, who had been in the armed services between 1963 and 1973, and had deployed to Vietnam.

The survey instrument built upon the one used in Waves 1 and 2, supplemented with additional measures (see below). We included a brief COVID-19 supplement to learn about coping with the pandemic and adoption/use of protective practices. The surveys were mailed with $5 enclosed and, upon completion, respondents could choose either to receive an additional $20 or donate it to The American Legion. The survey response rate was 74% (507 responded, 18 additional deaths were discovered, and 26 surveys were undeliverable). Among respondents, 422 men (83%) completed the COVID supplement and comprise the present sample.

### Measures

#### Combat

Combat was assessed at both Waves 1 and 2, using an eight-item Likert scale with five response options (*never* to *very often)*, yielding a total score ranging from 8 to 40 (3, 30, 31). This measure is highly internally consistent (Cronbach's alpha: 0.96, Wave 1, 0.94 Wave 2), with 0.88 test-retest reliability (32). Combat scores were categorized as low (8-15), medium (16-25), or high (26-40). In the present analysis we used combat exposure as reported in 1984 (Wave 1), since it was closer in time to the event. The 1998 combat score was used for 25 men who had missing items in their 1984 responses. Five veterans without combat scores at either wave were excluded from analyses involving combat exposure.

#### PTSD Symptoms

To maximize comparability, the same 18-item PTSD measure was administered at all three waves (33). This scale assessed symptom frequency within the past month, using a 5-point response scale, ranging from *Never* to *Very Often*. Items are consistent with the PTSD diagnostic criteria defined by the *Diagnostic and Statistical Manual for Mental Disorders – Version III* (34, 35). Reliability (Cronbach's alpha) was excellent at each wave (0.93 in 1984, 0.95 in 1998, and 0.96 in 2020).

In these analyses, we used the 2020 PTSD data to compute a total score (sum of responses, ranging from 18 – 90) (26, 33). Of the 507 veterans who responded in Wave 3, 20 (4%) did not complete all 18 items at Wave 1. For the 14 men who omitted only one item, and the six that omitted two, we imputed their PTSD scores by substituting the mean of the person-specific mean of 16 or 17 completed items and then summing all 18 items. Using these imputed items for the 20 men increased the mean PTSD score of the sample by <0.01%.

#### Later-Adulthood Trauma Reengagement

Late-onset stress symptomatology (also known as Later-Adulthood Trauma Reengagement) has been described as a phenomenon of older combat veterans who “experience increased combat-related thoughts, feelings, and reminiscences” that emerge as they get older and suffer age-related stressors/trauma/events (36). We used the 11-item short-form (LOSS-SF) of the 33-item scale originally developed by King et al. (37) to assess this phenomenon. Psychometric properties of the LOSS-SF have been described by Brady et al. (38). The LOSS-SF scale, which is the sum of the 11 items, was highly reliable in our sample (Cronbach's alpha = 0.95). For some analyses of social and preventive practices we categorized the LOSS-SF scale into low [11-24], medium [25-35] and high [36-54] tertiles and calculated odds ratios and trends.

#### Coping With COVID-19

Respondents were asked whether their military experiences had affected their ability to cope with “the COVID-19 situation” (made it *better, worse, both better and worse*, or *no impact*). Analyses compared any effect (better, worse, or both) to no effect. We conducted logistic regression analyses of this coping variable using Wave 1 combat scores, Wave 1 and Wave 3 PTSD symptom levels, and Wave 3 LOSS-SF scores as predictors. All respondents were born within a narrow range of years, so we did not control for age.

#### Open-Ended Responses

Respondents were also provided an open-ended option to further explain how their military experiences may have “helped or hindered in this situation.” The open-ended question was first coded as yes/no (140 and 282, respectively) to having provided a response. Next, among those who responded, responses were categorized into one of five mutually exclusive categories devised by one author (JMS) and verified by a second (SDS). Discrepancies in scoring were resolved through discussion. The categories were: (1) the military taught them to be disciplined, obey directives and be patient e.g., “Understanding the importance of following the rules” and “hurry up and wait”. (*discipline-patience*); (2) they learned to cope, e.g., “It is what it is.” (*coping*); (3) they explicitly mentioned an emotional need or PTSD, rather than an explicit military experience, e.g., “I have PTSD” (*emotional*); (4) they stated that experiences had neither helped nor hindered (*no effect*); (5) they made a negative or political comment, e.g., “Keeps my anger from the government and news media alive” (*political*). *T*-tests were used to compare the mean values for PTSD and LOSS-SF measures between those who did and did not provide responses in each category.

#### Preventive Practices

Men were asked whether they engaged in preventive practices regarding COVID. There were three yes/no questions on adoption of *social preventive practices* (social distancing, staying home, carrying out activities as usual) and five three-point response items (*not at all, occasionally*, or *frequently*) on *personal preventive* practices (handwashing, sanitizers, wearing masks, wearing gloves, disinfecting surfaces). Analyses of the personal practices contrasted those responding *not at all* or *occasionally* with those responding *frequently*. Because glove wearing was infrequently practiced, it was not analyzed.

#### Statistical Analysis

Analyses were conducted using SPSS v. 28. Pearson correlations were calculated, means of continuous variables were compared using *t*-tests, distributions were compared using chi-square tests and analysis of variance, and odds ratios were computed using logistic regression.

The study was approved by the Institutional Review Board of Columbia University.

## Results

Sample characteristics are described in Table 1. By design, the age range at Wave 3 was confined to the interval from 67 to 75 years (born 1945-1953; mean 72.5, standard deviation 1.6). Age is thus not considered to be a likely confounder. The men spent on average 2.8 years in the military (1 year in Vietnam) with a median Vietnam deployment date of August, 1968. The great majority were thus present during the period of peak combat intensity; accordingly, nearly two-thirds (64.3%) experienced medium or heavy combat based on our validated combat scale. It is therefore unsurprising that at baseline (1984), about one-third of the veterans (32.9%) had PTSD scores of at least 49, based on our 18-item PTSD symptom scale (range 18–90). Furthermore, the mean PTSD score increased in a dose-dependent manner with combat exposure, with the heaviest combat veterans scoring 50.9 points on average. Wave 3 introduced the LOSS-SF measure. Its mean score was 29.6 (SD 9.8), and it similarly increased with combat in a dose-dependent manner. There were no significant differences in any of the foregoing variables between those who did and did not return the COVID supplement, except that income reported in 2020 was significantly higher in those who did not return the supplement.

**Table 1:** Characteristics of 422 study respondents who completed the COVID supplement to the Wave 3 Survey^*^.

Average age at Wave 3, Mean ± SD	72.5 ± 1.6
Median month/year began military service	October, 1967
Average no. years in military	2.8
Median month/year deployed to Vietnam	August, 1968
Average no. years spent in Vietnam	1.0
Highest level of education	%
High school graduate or less	36.5
Some college	23.4
Vocational/technical	21.2
College graduate or higher	18.9
Income reported for 2019*	%
Under $25,000	8.2
$25,000–$49,999	43.1
$50,000–$99,999	38.8
$100,000–higher	9.9
Military combat in Vietnam, range 8–40	%
Low (8–15)	35.7
Medium (16–25)	43.9
High (26–40)	20.4
PTSD symptom score, Wave 1	
Percent with score ≥49	32.9%
Total symptom score, mean ± SD	43.3 ± 13.1
PTSD symptom score, wave 1, by level of combat, mean ± SD	
Low (8–15)	36.9 ± 10.8
Medium (16–25)	45.1 ± 11.6
High (26–40)	50.9 ± 14.5
LOSS-SF score (range 11–55) mean ± SD	29.6 ± 9.8
By PTSD symptom score at wave 1	
Low (18–36)	25.9 ± 9.4
Medium (37–48)	30.7 ± 10.0
High (49–90)	34.0 ± 9.8

* *There were no significant differences between those who completed the COVID supplement (n = 422) and those who did not (n = 85), except that a greater percentage of non-completers reported 2019 incomes of $50,000 or greater (62.2 vs. 48.7%, p < 0.05)*.

Military combat was strongly associated with perceived ability to cope with COVID-19. Just over half (51.3%) of veterans with high combat scores responded that their military experience affected their ability to cope with the pandemic, compared to 26.8% of those with low combat scores (OR = 2.9, 95% confidence interval [CI] 1.61–5.14). Since PTSD is clearly related to combat exposure in this cohort (8, 33), we used logistic regression to estimate odds ratios for association of Wave 1 and Wave 3 PTSD symptom scores with perceived coping ability as a binary (yes/no) outcome, as described above (Table 2). Taken separately, PTSD measured at Wave 1 and at Wave 3 were both significant predictors of perceived coping ability; however, the Wave 1 PTSD score is correlated with both PTSD at Wave 3 (ρ = 0.56) and LOSS-SF (ρ = 0.40), leading to Wave 1 PTSD no longer being a significant predictor in multivariate regressions that included Wave 3 PTSD with or without LOSS-SF (Table 2). The odds ratio for association of the highest level of Wave 3 PTSD with perceived coping, adjusted for LOSS-SF, was 2.44 (95% CI 1.11–5.37) (Table 2).

**Table 2:** PTSD and Late Onset Stress Symptomatology (LOSS-SF) scores as predictors of whether military experience affected coping with COVID.

**Model no**.	**Predictor(s)**		**OR (95% confidence interval)**
1	LOSS-SF		1.09 (1.07–1.12)
2	PTSD at Wave 1	Low	Ref.
		Medium	1.10 (0.61–1.98)
		High	1.37 (0.74–2.52)
	LOSS-SF		1.09 (1.06–1.12)
3	PTSD at Wave 3	Low	Ref.
		Medium	2.12 (1.07–4.17)
		High	2.44 (1.11–5.37)
	LOSS-SF		1.07 (1.03–1.12)

*Odds ratios (ORs) and 95% confidence intervals for association of scores for LOSS-SF, PTSD at Wave 1 (1984), and PTSD at Wave 3 (2020), using logistic regression with coping as binary outcome, and ‘no impact' as reference*.

*LOSS-SF is measured on a continuous scale, range 11–55. PTSD is categorical as Low (18–36), Medium (37–48), or High (49–90)*.

One-third (33.8%) of veterans who completed the COVID supplement also provided a response to the open-ended coping question (Table 3). Veterans who reported that their military experience affected coping were far more likely to provide an open-ended response than those who said their coping was unaffected (OR = 12.1, 95% CI 7.4–20.0). Veterans with PTSD symptom levels ≥49 were more likely to provide a response. Those who commented had an average Wave 3 PTSD score 8.3 points greater than those who did not (47.0 vs. 38.8, *p* < 0.001). Veterans whose responses were categorized as “emotional” had an average Wave 3 PTSD score 11.8 points greater than those with other types of responses (56.8 vs. 45.0, *p* = 0.001). Those whose responses were categorized as “political” had an average Wave 3 PTSD score 9 points greater than those with other types of comments (55.5 vs. 46.5), although this difference was not statistically significant. Veterans whose responses were categorized as “no effect” had an average Wave 3 PTSD score 9.8 points lower than those with other types of comments (38.0 vs. 47.8, *p* < 0.05).

**Table 3:** Mean scores (SD) of PTSD and LOSS-SF at Wave 3 for veterans who responded to the open-ended question “Please explain how your military experiences have helped or hindered you in dealing with this situation [COVID-19]”.

		**PTSD score Wave 3**				**LOSS-SF score Wave 3**		
	***N***	**Mean**	**(SD)**	***p*** **-value**	***N***	**Mean**	**(SD)**	***p*** **-value**
Any open-ended response	132	47.0	(14.5)	^***^	114	33.3	(9.4)	^***^
No open-ended response	259	38.8	(14.1)		240	27.7	(9.4)	
Discipline and patience								
Yes	43	46.2	(13.0)	n.s.	39	32.7	(9.2)	n.s.
No^§^	89	47.4	(15.2)		75	33.5	(9.6)	
Emotional								
Yes	22	56.8	(13.9)	^***^	17	40.9	(6.6)	^***^
No^§^	110	45.0	(13.8)		97	31.9	(9.2)	
No Effect								
Yes	11	38.0	(10.9)	^*^	12	29.3	(10.8)	n.s.
No^§^	121	47.8	(14.5)		102	33.7	(9.2)	
Political								
Yes	7	55.5	(11.9)	n.s.	5	37.2	(9.0)	n.s.
No^§^	125	46.5	(14.5)		109	33.1	(9.5)	
Coping								
Yes	30	43.5	(14.8)	n.s.	26	33.4	(8.0)	n.s.
No^§^	102	48.0	(14.3)		88	33.2	(9.9)	

* *p < 0.05*,

*** *p < 0.001, n.s., not significant*.

§ *Among veterans who provided at least one open-ended response*.

Veterans with higher LOSS-SF scores were also more likely to comment (Table 3). Those who provided a comment had an average LOSS-SF score 5.6 points greater than those who did not (33.3 vs. 27.7, *p* < 0.001), while those whose comments were “emotional” had an average LOSS-SF score 9 points higher than those with other types of comments (40.9 vs. 31.9, *p* < 0.001).

### Social Preventive Practices

Few veterans in this sample engaged in social preventive practices (Table 4): 4% practiced social distancing, 16.6% stayed at home; 32.3% went about usual activities. However, staying at home varied with Vietnam combat experiences (high combat = 5.7%, medium = 17.8%, low = 20.9%), with high-combat veterans significantly less likely to stay at home compared to low-combat veterans (OR = 0.23, 95% CI 0.08–0.68). Combat was not associated with either social distancing or pursuing usual activities. As shown in Table 4, veterans who stayed home had lower mean scores than veterans who did not stay at home both for PTSD (37.5 vs. 42.5, *p* < 0.05) and for LOSS-SF (26.6 vs. 30.1, *p* < 0.05).

**Table 4:** Mean scores (SD) of PTSD at Wave 3 and LOSS-SF according to social and personal preventive practices.

	**PTSD score Wave 3**				**LOSS-SF score Wave 3**			
	***N***	**Mean**	**(SD)**	***p*** **-value**	***N***	**Mean**	**(SD)**	***p*** **-value**
**Social preventive practices**								
Social distancing								
No	361	41.6	(14.8)	n.s.	327	29.7	(9.7)	n.s.
Yes	15	45.0	(14.5)		15	30.9	(10.8)	
Staying at home								
No	294	42.5	(14.7)	<0.05	261	30.1	(9.6)	<0.05
Yes	52	37.5	(13.2)		54	26.6	(9.7)	
Activities as usual								
No	95	39.6	(13.7)	n.s.	89	28.1	(9.9)	n.s.
Yes	208	42.4	(15.4)		190	29.7	(10.0)	
**Personal preventive practices**								
Handwashing								
No	56	34.3	(12.5)	<0.001	50	24.6	(9.4)	<0.001
Yes	330	42.7	(14.8)		302	30.4	(9.7)	
Using sanitizer								
No	150	39.7	(14.2)	<0.05	142	28.0	(9.7)	<0.01
Yes	218	43.4	(15.2)		192	31.1	(9.7)	
Wearing mask								
No	60	38.4	(13.7)	n.s.	59	26.3	(9.2)	<0.01
Yes	318	42.3	(15.0)		286	30.3	(9.8)	
Disinfecting surfaces								
No	201	39.3	(14.1)	<0.001	187	27.9	(9.4)	<0.01
Yes	151	45.0	(15.5)		134	32.4	(9.7)	

### Personal Preventive Practices

A large percentage of the veterans reported never or rarely using sanitizers (41.5%) or disinfecting surfaces (57.9%). Handwashing and mask-wearing were practiced frequently (85.5% and 83.0%, respectively). Wave 3 PTSD symptom and LOSS-SF scores were greater in veterans who frequently engaged in all four personal protective practices (handwashing, using sanitizers, wearing masks, disinfecting surfaces). The differences were statistically significant for all LOSS-SF scores, and for all PTSD scores, with the exception of mask-wearing (Table 4).

## Discussion

For the men in our sample, and for the great majority of those serving in US armed forces during the Vietnam Era, military service occurred in young adulthood. For many, the strict training and discipline associated with their military service appear to have intersected with their experiences of daily living during the pandemic in both positive and negative ways.

Using a sample of 422 veterans who had been deployed to Vietnam and had responded to previous surveys in 1984 and 1998 and to the COVID supplement in 2020, we examined the impact of their military experience on coping with the COVID-19 pandemic. We also examined whether combat exposure, later-adulthood trauma reengagement, and current PTSD symptom levels were related to their adoption of social and personal preventive practices. Although all respondents had served in Vietnam, their combat experiences varied greatly: 36% had little or no combat exposure while 20% experienced heavy combat. A half-century after their return from Vietnam, the mean PTSD score for the high-combat group reflected clinically significant PTSD symptomatology.

Deployed veterans who experienced medium or high combat exposure more frequently practiced personal protective behaviors, analogous to Sixsmith and co-workers' observations of British Second World War survivors whose wartime and subsequent life experiences had taught them to be self-sufficient and “maintain the level of cleanliness expected of him during his time in the Royal Navy… [and] continue to negotiate and structure their practical lives: managing, resilience and adaptability, and independence” (23). Indeed, about one-third of the open-ended responses in our study fell into the *discipline-patience* category (quoted here verbatim):

“In the Army taking orders what to do and not to do is about. Do as your told and listen makes life continue.”
“Understanding the importance of following the rules.”
“Hurry up and wait- standing in line.”
“This situation called for strict adherance to the rules for mask wearing, hand washing, distancing, etc. It had to be done and training made me more aware of consequences.”

Similarly, another third of those answering referred explicitly to what the military had taught them:

“Military service groomed me in my ability to cope with covid-19 stress. I am able to deal with following gov't directions easier.”
“The self discipline to stay home etc. Also to know that the war isn't won with just one battle.”

The scores reflecting later-adulthood trauma reengagement were significantly related only to having responded at all to the open-ended question and to the “emotional” category and not the categories related to politics and learned behaviors like discipline and patience. Many of the respondents specifically hearkened back to their days in Vietnam.

“Brought back my basic experience of confinement where I observed people around the best taking part in activities and coming and going as they pleased and I was not allowed out of the training are. I don't like confinement.”
“Tire of death and sickness”
“I was in a bad place, as for difficult place to cope for about 10 months. I seen a lot of experiences about 10–12 bad situations”
“Medical conditions from Agent Orange exposure made it hard to recover from COVID-19”

When the present survey was fielded, COVID-19 had moved beyond the northeastern states into the rest of the United States. Mask-wearing had been mandated in nearly all states where the respondents resided (39), which may explain why mask-wearing is the only social practice in which we observed no significant differences in adoption among combat exposure groups.

The absence of significant differences across combat categories in the qualitative responses supports the contention that the military experience itself provides veterans with positive life experience that can help them through potential adversity and challenges in their post-military lives. By contrast, the magnitude of difference in PTSD between those providing responses and those who did not fill in a response, and the fact that nearly 20% of responses to the open-ended coping question dealt with the veterans' own emotional health and not with their military experiences, as queried, provides strong support for the negative aspects of the military experience that as Gerber noted, are still powerfully present in many veterans (1). The few men who offered *no effect* comments (their military neither helped nor hindered them in the COVID situation) did not differ significantly from those who did not provide a comment. Perhaps they were less affected by traumatic exposures while in the military, and as such their military experiences were not as salient in the context of their current lives.

As reported in our earlier survey waves, (26, 33) and in many other studies of Vietnam veterans, combat exposure is significantly related to risk for PTSD symptomatology. Our quantitative data add further evidence that Vietnam veterans carry a special burden affecting their responses to the COVID-19 pandemic. We combined the three coping responses (*easier, more difficult* and *both easier and more difficult*) into a dichotomous any effect/no effect variable, positing that military experiences contribute to both positive and negative coping. The observation that veterans with medium and heavy combat exposure were more likely to respond that their military experiences had affected their ability to cope with COVID is consistent with Sixsmith et al.'s observation that “we tend to investigate wartime experiences through a partial [negative] lens.…Wartime experiences and the lives of older people [contain] both positive and negative connotations.” Further, it is noteworthy that those with higher levels of PTSD symptoms and trauma reengagement were also more likely to report that their military experiences impacted their ability to cope with the pandemic. Together, these findings underscore the lasting implications of wartime experiences, and are consistent with prior studies showing that trauma histories and related sequelae have implications for coping with future threatening situations (12, 13).

The finding between PTSD severity and staying-at-home behavior is somewhat counter-intuitive. One explanation may be that older veterans with PTSD often report that they try to remain engaged as they report experiencing increased intrusive memories and distress during quieter times when less is going on (22) – which may relate to less stay-at-home behavior. In the words of our respondents:

“I stay busy”
“I miss the interaction with fellow Vietnam Veterans, especially with my civilian friend. Its just not the same.”
“Isolation from friends and family members especially grandchildren”

This is also consistent with our work on the importance of social ties during physical distancing (22). As Gerber posits, perhaps “unit cohesion” (transmuting support from one's military unit onto one's family unit) could also help with trauma-related symptoms (1).

Others have observed a relationship between COVID-19 related behaviors and PTSD. Haderlein et al. reported that veterans with a clinical diagnosis of PTSD were more likely to receive a COVID-19 test, but were less likely to test positive, which raises the possibility that veterans with PTSD may be perceiving more risk and hence availing themselves more of testing opportunities (17).

We found that higher PTSD scores were significantly associated with handwashing and disinfecting surfaces, perhaps in response to increased perceived risk. This adherence to COVID-19 preventive practices is an interesting contrast to the group's smoking and drinking habits, where we have consistently observed elevated rates of these habits among those with higher rates of combat and PTSD (40, 41). Similar findings have been observed in other veteran groups (42). One possible explanation is that handwashing/sanitizers etc. are a kind of drummed in ‘military regimented' response that was learned in the service under combat, and that has translated itself to the new dangerous environment in which the veterans found themselves. Two veterans stated, for example:

“The safety washing hand and wearing a mask, taking orders from the Governor, as he wishes”
“Living day to day. Learning to respect senior decisions. Respect others as you want to be respected. Staying clean, to stay healthy. Self-discipline to wear masks where necessary and make every trip to town multi-purposeful. Difficulty- media coverage is politically directed as was the news from Vietnam making my decisions more difficult”

These findings support the importance of examining PTSD and potential health correlates across the life course (43). Also, despite disagreements about the risks, people perceiving greater risks were more likely to implement protective behaviors—especially later (vs. earlier) in March 2020. These findings have implications for risk communication (16).

In our PTSD scale 17 of the 18 items are nearly identical to those in the Posttraumatic Stress Disorder Checklist (PCL) based on DSM-IV (range 17–85) (44), with the additional question making the range of our measure 18–90. A PCL score of 44 is often considered indicative of a diagnosis of PTSD (45, 46). Our mean symptom score for high-combat respondents is 51.0 (± 14.5), and arguably consistent with a probable diagnosis of PTSD. We are currently examining the trajectory of the PTSD symptomatology in this group. Some of the symptoms in this aging population may be late onset. Whatever the course, a large number of men continue to carry psychological burdens related to their service in Vietnam. Our data may underestimate the extent of PTSD among all Vietnam veterans because participants are American Legionnaires who have joined a social organization despite possible PTSD symptoms. It is likely that some with PTSD would not be sufficiently high functioning or have the resources or emotional readiness to be involved in such an organization.

Veterans who endorsed thinking more about their military experiences and reengaging with trauma memories (reflected by higher scores on the LOSS-SF scale) were more likely to engage in personal preventive strategies. It is possible that those veterans who were engaged in a process of life review and making meaning of past military experiences were primed to consider their safety during the COVID-19 pandemic. At the same time, these veterans were less likely to engage in social preventive practices. This may be related to the nature of the later-adulthood trauma reengagement process – instead of avoiding people or situations due to perceived threat or trauma reminders, these veterans may actively seek out wartime friends and opportunites to talk with others about their military experiences. This process unfolds within a social context, and may partially explain these findings.

The associations between military trauma and PTSD symptomatology are well established, but the relationship between the psychological aftermath of deployment and war zone combat and subsequent reactions to new stressful or threatening situations has not been widely studied. Although more research is needed, clinicians and policymakers should be aware that these populations may be at special risk, especially since adherence to recommended public health practices is essential to controlling the COVID-19 pandemic and similar future emergencies. Veterans may benefit from programs and interventions designed to foster resilience in their personal lives and in the maintenance of relationships and support networks. Despite the passage of time, many veterans who served their country in Vietnam are, indeed, still burdened with “the things they carry” (47). A deeper understanding underlying dynamics of personal behaviors and reactions in this large group is thus an urgent need, as are expanded public health initiatives to provide assistance.

## Data Availability Statement

The datasets presented in this article are not readily available because our agreement with the American Legion to gain access to their private membership was that the data would not be shared. Requests to access the datasets should be directed to Jeanne M. Stellman, jms13@columbia.edu.

## Ethics Statement

The studies involving human participants were reviewed and approved by Institutional Review Board, Columbia University. Written informed consent for participation was not required for this study in accordance with the national legislation and the institutional requirements.

## Author Contributions

JS and SS created the original cohort, led the first two research waves, and they carried out the analyses. AS, APK, and BS contributed significantly to the modification of the Wave 1 and Wave 2 surveys and participated in conceptualization, analysis, and writing. All authors contributed to the article and approved the submitted version.

## Funding

The Foundation for Worker, Veteran and Environmental Health, Brooklyn NY, has funded all waves of this study and conducted this phase of the project. Data collection for Wave 1 was supported by The American Legion and the American Cancer Society. Data collection for Wave 2 was supported by the US National Academy of Sciences (subcontract NASVA-5124-98-001) (JS) and by U.S. Public Health Service grants CA-17613 (SS) and CA-68384 (SS). Additional support was provided by a VA Clinical Science Research and Development Service Senior Research Career Scientist Award (AS) and by VA Rehabilitation Research and Development (VA RR&D) Service Career Development Award IK2RX001832 (APK).

## Conflict of Interest

The authors declare that the research was conducted in the absence of any commercial or financial relationships that could be construed as a potential conflict of interest.

## Publisher's Note

All claims expressed in this article are solely those of the authors and do not necessarily represent those of their affiliated organizations, or those of the publisher, the editors and the reviewers. Any product that may be evaluated in this article, or claim that may be made by its manufacturer, is not guaranteed or endorsed by the publisher.
